# Novel Anti-Inflammatory Approaches for Cystic Fibrosis Lung Disease: Identification of Molecular Targets and Design of Innovative Therapies

**DOI:** 10.3389/fphar.2020.01096

**Published:** 2020-07-23

**Authors:** Christie Mitri, Zhengzhong Xu, Pauline Bardin, Harriet Corvol, Lhousseine Touqui, Olivier Tabary

**Affiliations:** ^1^ Sorbonne Université, Inserm, Centre de Recherche Saint-Antoine, CRSA, Paris, France; ^2^ Yangzhou University, Yangzhou, China; ^3^ Département de Pédiatrie Respiratoire, Hôpital Trousseau, AP-HP, Paris, France; ^4^ Equipe Mucoviscidose et Bronchopathies Chroniques, Département Santé Globale, Institut Pasteur, Paris, France

**Keywords:** cystic fibrosis, inflammation, anti-inflammatory, mucus, antibiotic

## Abstract

Cystic fibrosis (CF) is the most common genetic disorder among Caucasians, estimated to affect more than 70,000 people in the world. Severe and persistent bronchial inflammation and chronic bacterial infection, along with airway mucus obstruction, are hallmarks of CF lung disease and participate in its progression. Anti-inflammatory therapies are, therefore, of particular interest for CF lung disease. Furthermore, a better understanding of the molecular mechanisms involved in airway infection and inflammation in CF has led to the development of new therapeutic approaches that are currently under evaluation by clinical trials. These new strategies dedicated to CF inflammation are designed to treat different dysregulated aspects such as oxidative stress, cytokine secretion, and the targeting of dysregulated pathways. In this review, we summarize the current understanding of the cellular and molecular mechanisms that contribute to abnormal lung inflammation in CF, as well as the new anti-inflammatory strategies proposed to CF patients by exploring novel molecular targets and novel drug approaches.

## Introduction

Cystic fibrosis (CF) is the most common lethal monogenic disorder in Caucasians estimated to affect one out of 2.500-4.000 newborns. It is caused by a *Cystic Fibrosis Transmembrane conductance Regulator* (*CFTR*) gene mutation, which encodes a chloride channel expressed at the apical membrane of the epithelial cells (Riordan et al., 1989).

**Figure 1:**
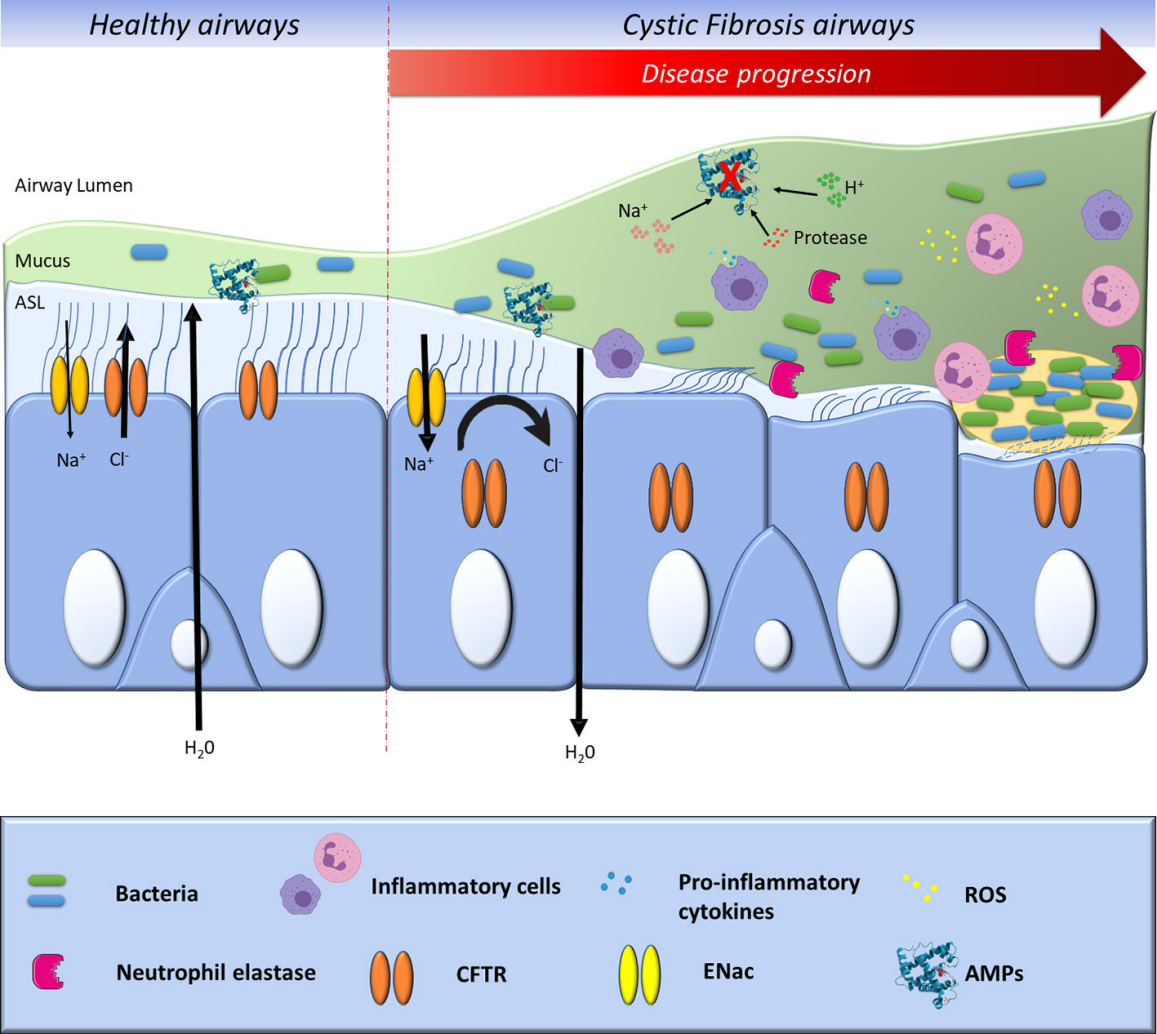
Progression of CF pathophysiology in bronchial epithelial cells. In healthy airways, sodium (Na^+^) absorption and chloride (Cl^−^) secretion control hydration of the airway surface layer (ASL). In CF airways, impaired Cl^−^ secretion due to the CFTR absence or loss of function leads to unregulated Na^+^ absorption and result in inadequate hydration of ASL, causing mucociliary clearance and bacterial killing impairment. As a result, mucus obstructs the lung airways and provides a nidus for bacterial infection and inflammation. The bacteria adhere to the surface and continue to grow, ultimately forming a biofilm. The inflammation of the CF lung is characterized by exaggerated secretion of pro-inflammatory cytokines by the airway epithelial cells, leading to the infiltration of polymorphonuclear neutrophils that release reactive oxygen species (ROS) and proteases. Neutrophil released elastase in the CF airway secretions correlates with lung function deterioration and respiratory exacerbations. The acidification of the ASL and the increase of its salt concentration, along with the increase of proteases levels, have been shown to impair the bactericidal activity of numbers of anti-microbial peptides (AMPs).

**Figure 2:**
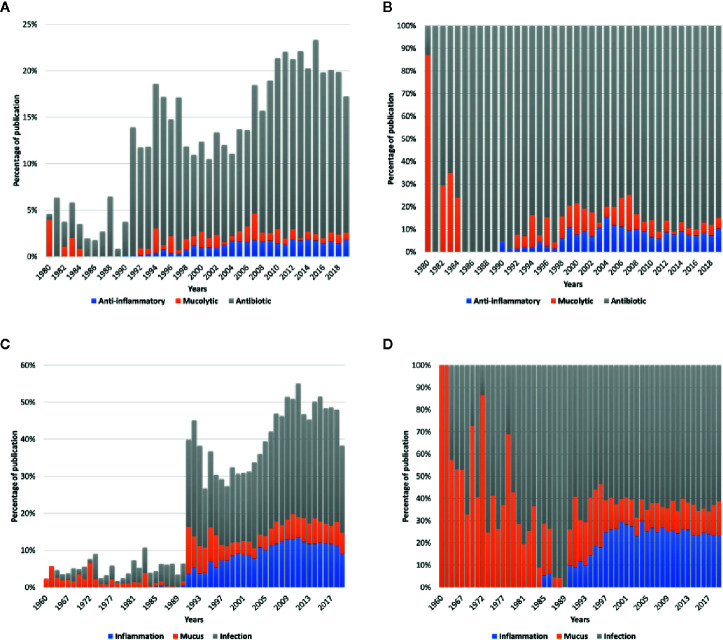
**(A)** The proportion of publications published in Pubmed (https://www.ncbi.nlm.nih.gov/pubmed) by years about “anti-inflammatory,” “mucolytic,” and “antibiotic” in combination with “cystic fibrosis” compared to the total number of publications in CF. **(B)** The proportion of publications published in Pubmed by years about “anti-inflammatory,” “mucolytic,” and “antibiotic” in CF. **(C)** The proportion of publications published in Pubmed by years about “mucus,” “infection,” and “inflammation” in combination with “cystic fibrosis” compared to the total number of publications in CF. **(D)** The proportion of publications published in Pubmed by years about “mucus,” “infection,” and “inflammation” in CF.

CF is a multi-system disease that affects the respiratory tract, intestines, pancreas, genital tract, the hepatobiliary system, and exocrine glands, leading to diverse pathology ranges and clinical problems (Elborn, 2016). While most patients have multiple organ alterations, the leading causes of both morbidity and mortality in more than 90% of patients remain chronic progressive pulmonary disease and respiratory failure (Elborn, 2016). In CF patients, the lack of CFTR chloride channel activity leads to progressive pulmonary obstruction associated with critical and constant neutrophil-dominated endobronchial inflammation and overwhelming bacterial infection (**Figure 1**). On a pulmonary level, scientists developed many new symptomatic therapies with either anti-inflammatory properties, antibiotics, or molecules improving mucociliary clearance (mucolytics) in order to treat inflammation, infection, or mucus abnormalities (**Figure 2A**). The discovery of these new drugs was made possible by the accumulation of knowledge in these three areas. After the discovery of CFTR, researchers aimed for the development of therapies that can correct the disease’s origin. Their work mainly focused on infection, rather than on anti-inflammatory drugs or mucus abnormalities. The proportion of published articles on infection is more than 70% compared to those published on inflammation or mucus. This proportion reaches more than 80% when focusing on publications on antibiotics compared to those on anti-inflammatory drugs and mucolytics (**Figures 2B, D**). In the allocation of priorities, the anti-inflammatory drugs have been, for long, the “poor relatives” in basic research compared to the modulators of CFTR activity.

These drug modulators targeting CFTR are designed to reestablish, at least partially, the CFTR expression, and improve its activity. So far, many of these treatments got through to the market, and these therapies are upgrading patients’ life quality through short- and long-term improvements in clinical outcomes (Lopes-Pacheco, 2019). Despite this, the main treatments remain symptomatic, focusing on different dysregulated clinical manifestations observed in CF patients (pancreatic insufficiency, intestinal malabsorption, and lung deterioration). However, their use is limited by insufficient basic scientific knowledge (**Figure 2C**), which has reduced the number of medicinal products currently on the market (Lopes-Pacheco, 2019). A deeper understanding of the natural evolution of CF pathology brought about new treatment tactics in order to improve pulmonary functions and increase life expectancy. CFTR chloride channel is also involved in the regulation of other channels such as the epithelial sodium channel (ENaC).

**Figure 3:**
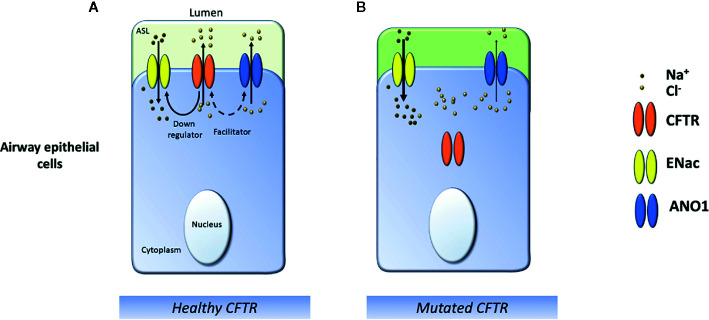
Schematic representation of ion transports in the cystic fibrosis airway. **(A)** In healthy airways, Na^+^ absorption, and CFTR and ANO1 Cl^−^ secretion regulate the hydration of the airway surface layer (ASL). Wild-type CFTR downregulates ENaC and participates in the activity of the ANO1 channel. **(B)** In CF airways epithelial cells, the lack of a functional CFTR channel reduces Cl^−^ secretion and causes Na^+^ hyperabsorption leading to ASL dehydration, which favors mucostasis.

**Figure 4:**
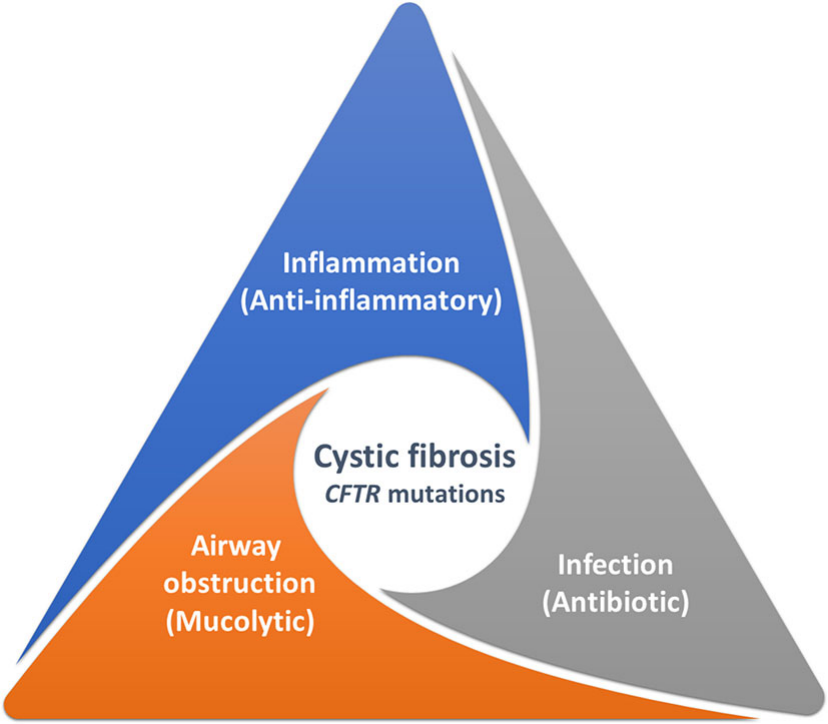
Interrelation between the main dysregulated aspects in the airway of Cystic Fibrosis patients. *CFTR* mutations affect inflammation, mucus properties, and infection. These different aspects are very intertwined, and treating one of these elements will have consequences on the other two.

Other channels are directly or indirectly linked to CFTR, such as the calcium-activated chloride channels ANO1 (also called TMEM16a) (Benedetto et al., 2017) (**Figure 3**). Therefore a deregulated CFTR activity leads to an abnormal mucus composition and alteration of the airway surface liquid (ASL) hydration that could participate in the inflammatory process in CF airways (Puthia et al., 2020). Recent publications have also highlighted that a loss of CFTR-mediated bicarbonate secretion and pH acidification impairs airways host defense by increasing mucus viscosity and reducing bacteria-killing (Shah et al., 2016). Current studies have established that the CFTR function is not restricted to ion transport regulation. Results have suggested a significant role of CFTR as a surface receptor for the internalization of *Pseudomonas aeruginosa* (*P. aeruginosa*) *via* endocytosis and consequent bacteria removal from the airway (Pier, 2000). In the CF airways, the permanent presence of bacteria might participate in the inflammatory process contributing to a vicious cycle between airway mucus obstruction, chronic infection, and exaggerated inflammation (**Figure 4**). Nowadays, it remains unclear how and why this vicious cycle is initiated, even though different elements suggest that different inflammatory pathways are deregulated in CF airways independently from infection (Bardin et al., 2019). However, mucus alterations could be one of the triggers of this process. Mucins tethering to the apical bronchial surfaces lead to acidification of ASL, thus reducing the anti-bacterial properties of CF airways (Song et al., 2006; Quinton, 2008; Adam et al., 2015).

Finally, it is essential to bear in mind that mucus alteration, infection, and inflammation are elements that are very carefully intertwined and difficult to separate in the process of an inflammatory response (**Figure 4**). Multiple hypotheses explain the early events leading to the CF lung pathophysiology progression (Jacquot et al., 2008a; Esther et al., 2019).

## Pathophysiology in CF Airways

### Inflammation

Although inflammation is a natural and protective process resulting from aggression, it plays a major role in CF lung pathology and progression. Inflammation was initially recorded by the Roman encyclopedist Aulus Cornelius Celsus in the 1^st^ century A.D. by some typical characteristic signs of inflammation as heat (calor), pain (dolor), redness (rubor), and swelling (tumor). Chronic and exaggerated inflammation in people with CF causes damages to lung tissues that can eventually lead to respiratory failure (Cantin et al., 2015). Many recent results show that bronchial epithelial cells play a significant role in the progression of the disease. In addition to being a physical barrier, epithelial cells secrete many inflammatory factors such as cytokines, eicosanoids, enzymes, and adhesion molecules (Roesch et al., 2018). This CF airway inflammation is characterized by an excessive production of interleukin (IL)-8 secreted by airway epithelial cells, and the presence of large numbers of neutrophils and macrophages among other inflammatory cells (Hubeau et al., 2001). However, it is not the only pro-inflammatory cytokine enhanced. In the airways of CF patients, TNF-α, IL-1β, IL-6, IL-8, IL-33, GM-CSF, and G-CSF are increased, also other molecules also play a major role such as the pro-inflammatory metabolites of arachidonic acid metabolism. Very recent results have highlighted the central role of other cytokines such as IL-17 (Roesch et al., 2018). In CF, the infiltration of inflammatory cells across the epithelium into the lumen can be very deleterious to epithelia and, as a consequence, requires robust regulation. Numerous works have tried to identify targets and strategies to reduce the exaggerated immune response that causes chronic inflammation without affecting the natural defenses against infection (Muhlebach and Noah, 2002). It is unclear whether the inflammation is a direct consequence of the cftr mutation or whether it is a consequence of infection and mucus accumulation. We do not know the contribution of infection to airway inflammation, but it must act as a catalyst and becomes self-perpetuating. Different studies have demonstrated the direct implication of the CFTR protein in this process mainly in the lung but also in extra-pulmonary tissues as the intestine or pancreas (Raia et al., 2000; Cohen and Prince, 2012; Stoltz et al., 2015; Bardin et al., 2019). Even before symptom onset, pulmonary inflammation and infection are often present in CF patients (Muhlebach and Noah, 2002). Although which comes first has been uncertain, this aspect is well reviewed in the article from Stoltz (Armstrong et al., 1995; Khan et al., 1995; Nixon et al., 2002; Stoltz et al., 2015). Moreover, new models lacking CFTR, including pigs, ferrets, and rat manifest inflammatory features typically observed with CF even in absence of infection (Rogers et al., 2008; Sun et al., 2010; Tuggle et al., 2014). For example, airways of CF piglets show no evidence of inflammation during the first hours after birth (Stoltz et al., 2010). Evidence has also demonstrated that non-infected human CF airway graft is in a pro-inflammatory state (Tirouvanziam et al., 2000; Tirouvanziam et al., 2002; Perez et al., 2007; Cantin et al., 2015). These data are reinforced by *in vitro* experiments using specific CFTR inhibitor. For example, Perez et al. have shown that Inh-172 treatment conducted in significant increase in IL-8 secretion in basal but also in response to *P. aeruginosa* infection (Perez et al., 2007). All these data support the hypothesis that mutations in cftr gene make epithelial cells intrinsically more pro-inﬂammatory compared with healthy cells (Perez et al., 2007; Cantin et al., 2015), which, once infection is introduced, sets the stage for mucosal damage and chronic airway infection (Tirouvanziam et al., 2000).

Although the link between CFTR deficiency and host inflammatory response remains unclear, this aspect has long been recognized as a central pathological feature, and consequently, an important therapeutic target. Some have hypothesized that in CF, the unfolded proteins accumulation on the endoplasmic reticulum induced a proteinopathy responsible for inflammation, impaired trafficking, altered metabolism, cholesterol, and lipids accumulation, and impaired autophagy at the cellular level. Some have speculated that chloride dysregulation participated in a stress-inducing ionic imbalance in the airway, with the implication of calcium activation, which could induce an inflammatory state (Ribeiro et al., 2005; Tabary et al., 2006a). New hypotheses have emerged with the direct activation of NOD-, LRR-, and pyrin domain-containing protein 3 (NLRP3) inflammasome and can be a key regulator of CF inflammation and a promising target (McElvaney et al., 2019; Jarosz-Griffiths et al., 2020).

However, since the appearance of high throughput sequencing, many studies have attempted to study the deregulated mechanisms, but the heterogeneity of samples and data makes analysis difficult. A meta-analysis of the different studies has summarized all this data (Ideozu et al., 2019). To summarize, many proteins are dysregulated, including gene from signal transduction (PI3K/Akt/mTOR signaling pathway) and immune system (NFκB and MAP kinase pathways), but this method is more relevant to highlight the consequence than the cause of the inflammatory dysregulation. A very recent article has confirmed the implication of NLRP3 inflammation activation due to the alteration of electrolyte homeostasis induced by the over-activation of β-ENac channel in CF (Scambler et al., 2019).

Furthermore, different authors showed more than 15 years ago that there is a deregulation of lipid metabolism in CF with an imbalance between pro-inflammatory metabolites of arachidonic acid metabolism and pro-resolving mediators form eicosanoid pathway (Freedman et al., 2004; Karp et al., 2004; Serhan, 2017; Roesch et al., 2018). Ceramide (CER) is an airway component composed of fatty acid and sphingosine that may alter the CF inflammatory response. CER is present in the cells membrane and when in contact with a specific stimulus, like a bacterial infection, CER in transmembrane signaling processes to help regulate cellular responses to infection by activating the inflammation processes. This could be an interesting alternative to treat CF inflammatory dysregulation by inhibiting CER synthesis (Mingione et al., 2020). Although there is no consensus regarding the regulation of CER in CF cells currently, even if more recent data have demonstrated their implication on the progression of CF lung disease (Horati et al., 2020; Mingione et al., 2020). Consequently, these results have led to the proposal that upregulated inflammation is related to the molecular defect of CF with a strong implication of nuclear factor kappa B (NFκB) or mitogen-activated protein (MAP) kinase pathways with other transcription factors including NFAT, NF-IL6, AP1 and AP2 (Tabary et al., 1999; Tabary et al., 2003; Muselet-Charlier et al., 2007).

More recently, different articles have also associated microRNA (miRNA) dysregulation to CF inflammation (Fabbri et al., 2014; Bardin et al., 2018a; Bardin et al., 2019). How the lack of CFTR expression in ionocytes, ciliated, and submucosal gland epithelial cells of the respiratory tract, boosts pulmonary inflammation is still partially comprehended. Different authors have also highlighted the central role of neutrophil in CF airway inflammation, and many believed that bronchiectasis results from the proteolytic and oxidative damage induced by these cells. Longitudinal data from the Australian Respiratory Early Surveillance Team for Cystic Fibrosis demonstrated that neutrophil elastase activity at 3 months of age was a predictor of bronchiectasis at 12 months and 3 years (Wijker et al., 2020). The central role of neutrophils and its genesis has been extensively review by Nichols et al. and Perrem et al. (Nichols and Chmiel, 2015; Perrem and Ratjen, 2019).

Understanding the initial host defense defects in CF airways could suggest new prevention strategies and treatments, the means to assess disease status and efficacy of therapeutics (Stoltz et al., 2015). Several mechanisms are suggested to explain in what way CF basal inﬂammation promotes subsequent bacterial infection. One possible explanation is that serine protease, released by activated neutrophils, degrades innate immune mechanisms, including anti-microbial peptides (AMP), participates in secondary infection, and to this vicious cycle. The molecular mechanisms relating to abnormal CFTR chloride function in airway epithelial cells to excessive lung neutrophilic inflammation have not yet been fully clarified even if extensive works have already been published (Taggart et al., 2000; Tabary et al., 2006b). Decreased neutrophil apoptosis and the high secretion of IL-8 by epithelial cells are contributing factors. In 2016, researchers discovered the leukocyte adhesion deficiency IV (LAD-IV), which is a defect in monocyte integrin activation in CF patients. The study showed that CFTR mutations could lead to a monocyte-specific adhesion deficiency (~80%) and impairment in transmigration into the alveolar space, which could explain the extreme infiltration of neutrophil since monocytes play a crucial part in inflammation and its resolution. Thus, failing to recruit monocytes in CF patients’ lungs may explain the excessive production of cytokines, the impaired inflammation resolution, and pathogen capture impairment (Sorio et al., 2016). The continuous driven recruitment of neutrophils and other immune cells and their implication in non-resolving inflammation have been already discussed in different reviews (Cantin et al., 2015; Nichols and Chmiel, 2015; Roesch et al., 2018).

Whether CFTR dysfunction causes directly or indirectly, a more important predisposition to infection and whether the inflammation occurs separately from the infection has yet to be determined. The development of new anti-inflammatory strategies in CF remains limited due to the limited researches in this area compared to infection (**Figure 2D**).

### Bacterial Infection

Respiratory infections in CF occur from childhood. In progressive lung diseases like CF, typical pathogens (*P. aeruginosa, Streptococcus aureus, Burkholderia cepacia, Achromobacter xylosoxidans*) colonize the airways (Palser et al., 2019). More than 50% of children diagnosed at birth have shown positive *P. aeruginosa* cultures by five years of age (Palser et al., 2019). If *P. aeruginosa* is neither spontaneously cleared nor eradicated with antibiotic therapy, the CF lung environment facilitates the infection.

The presence of pathogens triggers inflammatory processes in the airways contributing to the destruction of the cell barrier. Since inflammation is a natural process of defense and the eradication of pathogens, limiting it too much or for a long term could be counterproductive. For this reason, antibiotics are more frequently recommended than anti-inflammatory drugs in CF lung disease treatment and could indirectly serve to diminish airway inflammation (Oermann et al., 1999). The anti-inflammatory drugs that could alter the natural defense of the lung are only prescribed during exacerbations. Constant development and ideal usage of new anti-microbial compounds are vital for improving the CF patients survival chance and quality of life (Waters and Smyth, 2015). As a result of long-term antibiotic treatment, the decrease in infection and inflammation is associated with lung function improvements and pulmonary exacerbations reduction (Waters, 2018).

In a normal situation, the airways can defend themselves by forming a physical barrier between the outside and the inside. Also, the lung is capable of secreting cytokines that will allow the recruitment of inflammatory cells, but it is also capable of secreting anti-bacterial molecules. Thus, many natural AMPs, contained in the airways, are part of the innate immune response to the airway defense (Hancock et al., 2016). AMPs exhibit microbicidal activities on a broad spectrum of microbes, but bacteria appear to be the most targeted pathogens (Scott and Hancock, 2000; Zasloff, 2002). AMPs can kill bacteria rapidly in a few minutes. If most of the AMPs kill targeted pathogens *via* an electrostatic action on their membranes, some of them kill by more sophisticated mechanisms such as the IIA secretory phospholipase A2 (sPLA2-IIA) which kills bacteria through selective hydrolysis of their membrane phospholipids (Van Hensbergen et al., 2020), or by interfering with intracellular targets in bacteria (Geitani et al., 2019; Wang et al., 2019). Except for very few examples, little is known about the specificity of AMPs toward Gram-positive *vs.* Gram-negative bacteria. The sPLA2-IIA is one of the rare AMPs that target Gram-positive bacteria that exerts its bactericidal effect at much lower concentrations than other molecules [For details, see the review (Van Hensbergen et al., 2020)].

AMPs represent an essential part of the host defenses against infections and also as a potential therapeutic tool, as has been shown in infections animal models (Morrison et al., 2002; Piris-Gimenez et al., 2005). This effect was also supported by studies in patients with infectious diseases showing that altered AMP expression and/or gene polymorphisms were associated with increased infections (Rivas-Santiago et al., 2009). On the other hand, unfavorable circumstances for AMPs actions as abnormal salt concentration or acidification, and inactivation by proteases, in ASL of CF patients (**Figure 1**), have been shown to inactivate AMPs bactericidal functions which may explain increased airway infections (Bals et al., 2001; Lecaille et al., 2016; Simonin et al., 2019). Normalizing ASL pH by inhibition of the persistent proton secretion, mediated by ATPase H^+^/K^+^ transporting non-gastric alpha2 subunit (ATP12A), might enhance innate airway defense in CF newborns during the onset of *S. aureus* infection. A recent study showed that the hydrophobic N-terminal domain of *Cg-*BigDef1 (a big defensin from oysters) exhibits salt-stable interactions with bacterial membranes opening the doors to eventual drug developments when physiological salt concentrations inhibit the anti-microbial activity of β-defensins such as in CF disease (Loth et al., 2019).

In parallel to their anti-microbial functions, several AMPs have been shown to play immuno-modulatory roles, in particular by interacting with the inflammatory reaction produced by host cells. Several studies have shown that AMPs can target host cells involved in innate immunity and modulated their production of inflammatory mediators, including cytokines. Although it is not always easy to dissociate these actions as most AMPs exhibit both functions, depending on their concentrations, the host cell targets, and the environments. However, AMPs have been shown to impair the inflammatory reaction induced by invading pathogens by different mechanisms (Masera et al., 1996; Finlay and Hancock, 2004; McInturff et al., 2005).

The anti-inflammatory potential of AMPs correlates with their capability of attracting and recruiting neutrophils and other inflammatory cells. They may also have direct or indirect effects on their maturation, differentiation, degranulation, or apoptosis (Lai and Gallo, 2009). AMPs also act by blocking neutrophils apoptosis, therefore prolonging their lifetime, and ultimately their phagocytic functions (Nagaoka et al., 2012). AMPs can also potentiate the effects of inflammatory cells such as macrophages while limiting other tissue damage (Brook et al., 2016).

### Mucus Alteration

In healthy people, ASL is a thin liquid film covering the airways and participating in mucociliary clearance and airways desiccation (**Figure 1**). Historically, studies suggested that different secretory cells (goblet cells, submucosal glands cells, and serous cells) contribute to ASL production (Tarran et al., 2001). The recent finding of the airway “ionocyte” could similarly result in a revised understanding of ASL production (Plasschaert et al., 2018). This group has identified by RNA sequencing all the RNAs present inside airway cells and by a new method, called pulse-seq, has discovered this scarce cell type. They created the term “ionocytes” due to the cell’s likeness to ionocytes in charge of regulating ion transport and hydration in the fish gills and frog skin. In the airway, ASL consists of two main layers: 1) the apical layer consisting of a water-based polymeric mucus, and 2) a periciliary layer (PCL) that bathes the epithelium (Atanasova and Reznikov, 2019). Normal mucus is made of 97% water and 3% proteins, lipids, and salt. The mucus gel layer acts as a physical barrier to prevent most pathogens from accessing the cells (Button et al., 2012). The mucus hydration and the mucin concentration dramatically affects its viscoelastic properties, which, in turn, determines how effectively it is cleared from the distal airways toward the trachea by ciliary action and cough (Fahy and Dickey, 2010).

The commonly accepted explanation for airway disease in CF is the “low volume” hypothesis. A reduced volume of the periciliary fluid layer (PCL) causes failure of mucociliary clearance, the ‘lungs’ innate defense mechanism. In addition to having altered physical properties, the mucus composition is modified and will participate in the CF pathophysiology by altering host defense proteins (Henderson et al., 2014). An increase in mucin secretion and an abnormal composition of mucus are implied by the formation of endobronchial mucus plaques and plugs. Mucus present in bronchia becomes the primary site of airflow obstruction, and subsequently for chronic infection, and persistent inflammation leading to early small airways disease succeeded by bronchiectasis development. Increased mucus and airway obstruction are hallmark features of multiple respiratory diseases and contribute, especially in CF, to a complicated, inflammatory process (Puthia et al., 2020). A chronic cycle of infection and inflammation could be initiated, resulting in airways structural integrity damages and leading to bronchiectasis development (Chalmers et al., 2017). More recent studies from Esther *et al.* have shown that the increase of mucus burden and inflammatory markers without infection suggest that mucolytic therapies could serve as preventive therapy for CF lung pathology (Esther et al., 2019). More, mucus composition and properties also depend on the levels of mucin production by epithelial cells that can be increased by bacteria suggesting a complex role of inflammation, infection, and mucus, especially in CF pathology (Mohamed et al., 2012). The up-regulation of airway mucin genes by inflammatory/immune response mediators at the transcriptional and/or posttranscriptional level is one of the major contributors to mucin overproduction. The MUC5AC gene is transcriptionally up-regulated by several inflammatory mediators, including LPS, IL-9, neutrophil elastase, TNF-α, and IL-1β (Song et al., 2003). IL-8-induced binding of RNA-binding proteins to the 3-untranslated region of MUC5AC is a potential mechanism for regulating *MUC5AC* gene expression at the posttranscriptional level (Bautista et al., 2009). Several studies have shown that PMA induces a matrix metalloprotease-mediated release of transforming growth factor-(Shao et al., 2003). Eicosanoids mediate inflammation and mucus secretion in chronic pulmonary inflammatory diseases (Garcia-Verdugo et al., 2012). Some studies in the field have shown a substantial increase of eicosanoid levels, including PGE2 and LTB4 in CF airways (Bautista et al., 2009) and CF bronchial epithelial cells (BECs) stimulated by LPS from *P. aeruginosa* (Medjane et al., 2005). On the other hand, this bacterium stimulates mucus production through the induction of several mucins such as MUC5AC and MUC2 both in cultured BECs and in a mouse model of lung infection by *P. aeruginosa*. This induction mainly involves the stimulation of BECs by flagellin through the TLR5 and Naip pathways and is accompanied by the secretion of IL-8 by BECs, which amplify mucus production (Mohamed et al., 2012).

Thus, we can suggest that in CF airways, mucus abnormalities offer a niche that favors bacterial infections, which in turn amplify mucus accumulation *via* a vicious circle that can participate in the exacerbation of the severity of CF disease. This amplification can occur either directly *via* virulence factors (such as flagellin and LPS) of infecting bacteria or *via* cytokines and eicosanoids produced by CF airways during infection.

### Proteases and Lipids Imbalance

Current studies on mucolytic agents therapy used in CF have been demonstrated to increase markedly neutrophil elastase (NE) activity in CF sputum. Serine proteases, including NE, cathepsin G, and proteinase 3, are the three most major proteases found in the CF lung. These proteases are not only secreted by BECs, but also by monocytes, lymphocytes, granulocytes, and, more importantly, neutrophils (Pelaia et al., 2004; Hunt et al., 2020). Different approaches have exposed their participation in intracellular and extracellular activities, including inflammation, tissue remodeling, mucin expression, bacterial killing, and neutrophil chemotaxis. NE, a significant product of neutrophils granule degranulation, is extensively studied in CF and is implicated in cleavage and inactivation of CFTR protein (Chalmers et al., 2017). Besides, NE also upregulates IL-8 and participates in activating cysteinyl cathepsins and matrix metalloproteases.

In the CF airway, different articles have described the protease and anti-protease imbalance, which could be explained by two different mechanisms (Galli et al., 2012; Causer et al., 2020). Firstly, CFTR is also a transporter of glutathione (GSH), a protease that is the main non-enzymatic antioxidant present in the ASL (Rahman and MacNee, 2000). Antioxidants are an essential protective response to tissue injury and occur mainly in an inflammatory environment. An absence of GSH in the extracellular medium disequilibrates this balance and induces an oxidative environment. This environment is intended to fight bacteria and viruses that may be present. The goal of this process is to break up and eliminate the injured tissues and, thus, promote tissue repair for the inflammatory process resolution. When this natural response arises in an uncontrolled way, the outcome is extreme tissue damage that could induce chronic inflammation, as observed in CF (**Figure 1**). During inflammation, reactive oxygen species (ROS) such as the superoxide anion are liberated by phagocytes and are thought to be the main cause of tissue damage.

In CF, the presence of numerous inflammatory cells that release many oxidants will have a significant role in the deregulation of the pro- and anti-inflammatory balance. Lung cells are vulnerable to the damaging effects of ROS and release inflammatory mediators, thereby amplifying lung inflammation. ROS are extraordinarily reactive, and when produced near the cell membranes, they diminish intracellular GSH and cause lipid peroxidation, which may harshly disrupt its function and may lead to cell death or DNA damage in alveolar epithelial cells. So, when ROS production increases, the redox balance of the airways is altered, and this can lead to bronchial hyperactivity and to further inflammation and participates in CF co-morbidity. GSH is a sulfhydryl containing tripeptide (L-γ-glutamyl-L-cysteinyl-glycine) that scavenges oxidants and could, therefore, participate in the control of the inflammatory process by reducing oxidative stress (Rahman and MacNee, 2000; Ehre et al., 2019). Therefore, a CFTR deficiency leads to an increased accumulation of intracellular GSH in the epithelial lining fluid compared with plasma. Secondly, different dysregulated parameters such as infection, inflammation, and hypoxia, increase the free radicals derived from oxygen and nitrogen. This pro-oxidative environment may directly exert its effects by activating transcription factors such as NFκB and MAP kinase pathways responsible for the coordinated expression of numerous genes involved in inflammation, cell death, proliferation, as well as cytoprotection and antioxidant defenses (Pelaia et al., 2004).

CFTR-deficient tracheal epithelial cells are characterized by high GSH levels that decrease the intracellular content of ceramide (CER). CER deficiency occurring in CF seems to be responsible for the increased activation of the pro-inflammatory transcriptional nuclear factor NFκB that, in turn, is responsible for the abnormally high inflammatory response in CF respiratory epithelial cells (Vilela et al., 2006; Aureli et al., 2016). An increasing number of studies indicate that sphingolipids play an important regulatory role in CF concerning pulmonary inflammation. In different models, it has been shown that *de novo* sphingolipid synthesis is an inflammation responsive pathway. It is enhanced by inflammatory mediators, both at transcriptional and enzyme activity level, and the accumulation of its metabolite CER potentiates inflammation in a vicious circle (Caretti et al., 2014). Sphingosine-1-phosphate (S1P), generated in the nucleus by phosphorylation of SphK2 ((Sphingosine Kinase 2), modulates HDAC (histone deacetylases) activity either by direct binding or through activation of nuclear ROS, and, regulates cell cycle and pro-inflammatory gene expression (Fu et al., 2018). The accumulation of CER causes *Cftr* ^-^ deficient mice to suffer from constitutive age-dependent pulmonary inflammation, death of respiratory epithelial cells, deposits of DNA in bronchi, and high susceptibility to severe *P. aeruginosa* infections (Teichgräber et al., 2008). Aggregates accrual, formed by misfolded mutant CFTR and a miscellaneous of sequestered proteins within, induces inflammation and oxidative stress, impairing proteins and lipids transport, and consequently inflammatory statement (Mingione et al., 2020).

## History of “Classical” Anti-Inflammatory Drugs

A better understanding of the molecular mechanisms involved in inflammation has led to the development of new anti-inflammatory therapeutic strategies. In CF, the intertwining of inflammation, infection, and airway mucus obstruction complicates therapeutic approaches. Thus, anti-inflammatory treatments, combined with antibiotic therapies and airway clearance techniques, play an essential role in patient care, particularly during periods of exacerbations and hospitalization.

### Steroid Anti-Inflammatory Treatments

Glucocorticoids (GC), a class of corticosteroids (CS), are potent anti-inflammatory molecules frequently applied in the treatment of “inflammatory” pulmonary diseases. GC target many of the proteins involved in inflammation, including IL-1β and IL-8 and NFκB and activator protein (AP-1) (Tabary et al., 1998; Barnes, 2006). Until recently, CS were the main anti-inflammatory CF treatments and were mainly used during exacerbations through inhaled or oral administrations (Balfour-Lynn and Welch, 2014; Lands and Stanojevic, 2019).

Since the first Prednisone clinical trials (Auerbach et al., 1985), oral CS have been shown to diminish the lung inflammation and reduce the development of the pathology in CF patients. However, the use of CS is still controversial in the CF context due to medium- and long-term use. The side effects include growth impairment, cataract formation, glucose intolerance, and osteoporosis (Balfour-Lynn and Welch, 2014). Nonetheless, oral CS are promptly used during an exacerbation to decrease inflammation in CF lungs.

Even though the use of GC in CF is common, the signaling pathways remain partially described. Interestingly, we have published that the NFκB signaling pathway was significantly involved and refractory to the action of GC in glandular epithelial cells (Tabary et al., 1998). Moreover, we have confirmed these results in airway neutrophils from CF patients (Corvol et al., 2003).

Even though inhaled CS have a better safety profile, their efficacy has not yet been demonstrated (Balfour-Lynn and Welch, 2014). The inhaled steroids withdrawal impact was established in a multicentric randomized, double-blind placebo-controlled trial, including CF children and adults (Balfour-Lynn et al., 2006). This study failed to show any beneﬁcial eﬀect of inhaled CS in CF patients treated for six months.

Finally, GC remain interesting molecules, especially during exacerbations, as they significantly reduce inflammation. However, their use in CF can only be limited to specific cases.

### Non-Steroid Anti-Inﬂammatory Treatments

As GC have significant side effects, alternative molecules have been proposed. For a few years, Ibuprofen, a non-steroidal anti-inflammatory drug (NSAID), has emerged and was proposed to the CF patients as a GC alternative. Most of NSAID (such as Aspirin) are known to block cyclo-oxygenase (COX) enzymes that produce prostaglandins from free arachidonic acid (Kurumbail et al., 1996). Ibuprofen, discovered by Stewart Adams laboratory in 1961, was sold initially as Brufen to treat rheumatoid arthritis (Balfour-Lynn et al., 2006; Halford et al., 2012). In CF, Ibuprofen acts directly on neutrophil activation, inhibiting their mobility and recruitment in the airways (Konstan et al., 2003). High-dose of Ibuprofen can reduce the development of CF patients’ lung disease, especially in children (Lands and Stanojevic, 2007; Lands and Stanojevic, 2019). A meta-analysis from a current update of a regular review has been published on the Cochrane database (Lands and Stanojevic, 2019). Multiple unwanted effects were a matter of concern due to the high doses usage, which has limited the Ibuprofen use in CF. Recent results have described that obvious benefits of Ibuprofen therapy outbalance the low risk of gastrointestinal bleeding, although long-term safety results are limited. In low doses, some shreds of evidence indicate that Ibuprofen may cause inflammation (Lands and Stanojevic, 2019). Nonetheless, these outcomes are still a subject of debate among scientists who suspect the inappropriate use of Ibuprofen for CF patients (Lands and Stanojevic, 2016). The association of Ibuprofen with infections is more complicated in that it confers risk in some situations but benefits in others, therefore its usage might require close monitoring (Varrassi et al., 2020).

### Macrolides

Among the most exciting new anti-inflammatory drug treatments established in the last few years in the CF context the macrolides (Southern et al., 2012). Macrolides were discovered in 1952 and were initially isolated from cultures of *Streptomyces erythraea*. The frequently used macrolides have 14 (Clarithromycin, Erythromycin, and Roxithromycin) or 15 (Azithromycin) atoms attached to their macrocyclic rings and were named macrolides in regards to the presence of macrocyclic lactone ring. Macrolides are interesting original antibiotics because of their double action of not only reducing infections but also reducing inflammation. The macrolides were used as antibiotics to treat different infectious diseases, including numerous airway pathology as pneumonia, CF, bronchitis, pharyngitis (Zalewska-Kaszubska and Gorska, 2001). Surprisingly, in 1987, a Japanese group has reported a spectacular effect in panbronchiolitis patients’ lifespan when treated with Erythromycin antibiotic (Kudoh et al., 1987). This pathology is a typical and representative disease of chronic respiratory tract infection in Japan, characterized by chronic inflammation localized predominantly in the respiratory bronchioles with inflammatory cells such as monocytes, macrophages, neutrophils and, T lymphocytes.

The molecule showing the most interesting effects in CF patients is Azithromycin, with an improvement of lung parameters, a decrease of *P. aeruginosa* infection, and hospitalization duration (Clement et al., 2006; Saiman et al., 2010; Nichols et al., 2020). Prolonged use of small dose Azithromycin was related to a beneficial impact on lung disease expression, well ahead of *P. aeruginosa* infection. A metanalysis of these researches proved substantial improvement or maintenance of the forced expiratory volume in one second (FEV1, a measure of lung function) and forced vital capacity (FVC) in treated patients *vs.* controls after 12 months of therapy. Even though there was no decline in the intravenous antibiotic therapy necessity or the hospitalization duration of any of these studies, a positive effect on the restoration of Cl^-^ efflux in CF has also been shown (Saint-Criq et al., 2011).

Moreover, some scientists demonstrated that macrolides operate by limiting pro-inflammatory cytokines and provoking direct alterations in the neutrophils function (Equi et al., 2002; Southern and Barker, 2004; Haydar et al., 2019). However, they failed to reduce the inflammation in BECs in CF patients (Saint-Criq et al., 2012). One recently published article has demonstrated that Azithromycin could modify the M2 phenotype macrophage and, therefore, indirectly modify the inflammatory process by inhibiting NFκB activation by increasing IKKβ expression in J774 murine macrophages (Haydar et al., 2019).

However, some macrolides, such as Clarithromycin, can induce neutrophil extracellular trap (NET) generation, a mechanism implicated in innate immunity and some inflammatory processes. NETosis is a mechanism by which neutrophils extrude their DNA and protein contents to form NET, including AMPs. The physiology and the formation of the NET have been extensively described in the review from Ravindran et al. (2019). In the fetal stage and early childhood, neutrophilic inflammation in the peri-bronchial regions is present in CF patients who have mucus excess and obstructive secretions but no persistent bacterial infections. Various microbial components like inflammatory cytokines, lipid mediators, and extracellular DNA found in CF patients induce NET formation (Henke and Ratjen, 2007). In CF airways, neutrophils are recruited to the airway upon infection and exacerbate the disease by producing NETs, which can increase mucus viscosity and consequently participate in the airway obstruction. The excess of NETs and their cytotoxic components, associated with hypervisquous mucus, exacerbate CF NET produced by Clarithromycin and inhibit *Acinetobacter baumannii* infection by acting on its growth and biofilm formation in an LL-37-dependent manner (Konstantinidis et al., 2016; Khan et al., 2019). Clarithromycin also enhances the antibacterial defense of fibroblasts and improves their wound healing capacity through the upregulation of LL-37 on NET structures (Arampatzioglou et al., 2018). Although Azithromycin and Chloramphenicol show that neutrophils pretreatment with these macrolides decreases the NETs release. Moreover, Azithromycin showed a concentration-dependent effect on respiratory burst in neutrophils, whereas Chloramphenicol did not affect degranulation, apoptosis or respiratory burst. So, these antibiotics modulate the ability of neutrophils to release NETs influencing human innate immunity (Bystrzycka et al., 2017). The macrolide immunomodulatory role depends on the macrolide used and the pathology involved.

As a final point, conventional anti-inflammatory treatments for CF are limited and have not been explicitly developed for this pathology, and could induce counterproductive effects. Research in this field is still limited compared to antibiotics, but despite this, new molecules or strategies are being evaluated.

## Novel Anti-Inflammatory Approaches

Better insight into the pathways involved has led to the development of new therapeutic approaches that are currently being evaluated under cell experiments or clinical trials. These new strategies aiming at the CF inflammation are designed to treat different dysregulated aspects such as channel modulators, oxidative stress, cytokines secretion, lung remodeling, and the regulation of dysregulated pathways.

### New Channel Modulators

#### CFTR Channel

The discovery of the *CFTR* gene in 1989 resulted in insights on how CFTR mutations induce CF pathology and encouraged many researchers to develop new drugs or strategies to correct the mutation or increase the protein activity (Riordan et al., 1989). Genetic therapy using adeno-associated virus (AAV) or other strategies aiming to correct the CFTR gene was very promising because CF is a monogenic disease. Nonetheless, the subsequent realization tempered expectations because the airways are well defended and are not absorptive surfaces. The natural barrier of mucus considerably impairs gene transfer into the lungs, and the epithelium renewing necessitates numerous administrations. For these reasons, only one study has demonstrated a significant but moderate effect on CF patients. Thus, further optimizations or other strategies are needed and in progress (Alton et al., 2015; Alton et al., 2017).

**Figure 5:**
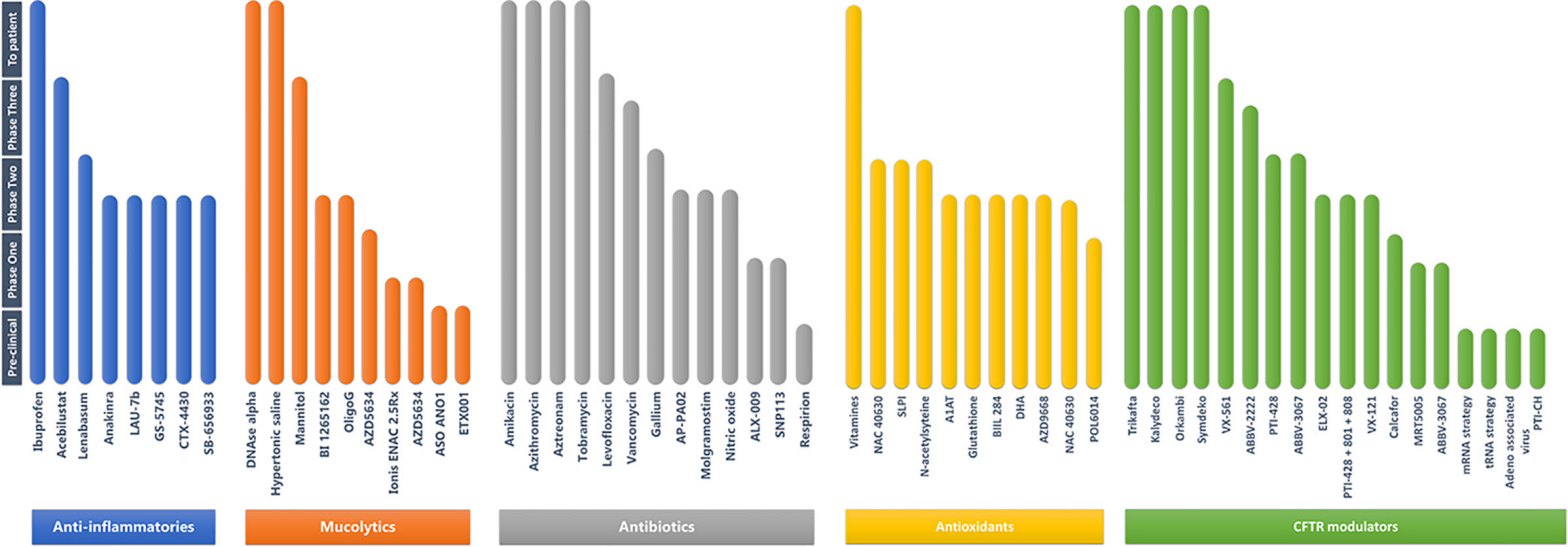
List of the different categories of drugs under development or clinical trials in the context of CF (adapted from https://www.cff.org/Trials/pipeline/).

**Figure 6:**
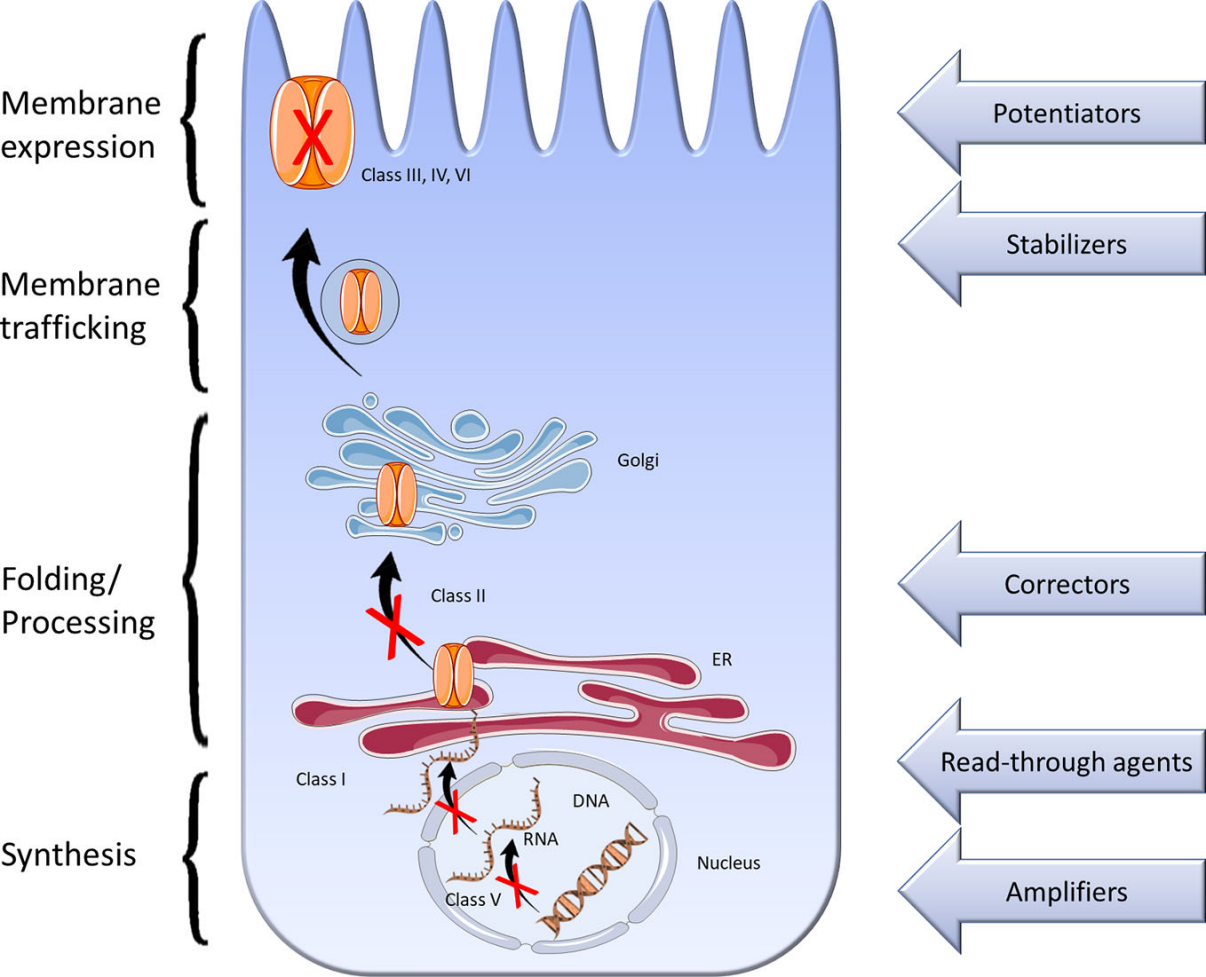
Description of the different classes of CFTR mutations related to the different therapeutic proposed in the literature. I—ynthesis defect, II—processing defect, III—channel gating defect, IV—channel conductance defect, V—reduced CFTR production, VI—defect of stability; ER, endoplasmic reticulum.

These data provided the grounds for pharmacologic modulations of chloride transport, by targeting mutant CFTR and/or alternative ion channels as anoctamin-1 (ANO1) that can compensate for CFTR malfunction. This excitement has now proven to be warranted because numerous new therapies approved by the FDA or EMA are now either in the pipeline or available for CF patients (**Figure 5**). This finding contributes to the innovation of genetic disease pharmacotherapy with *Vertex Pharmaceuticals* as a leader in the CF research field. Fundamental CF research has set the stage for a better molecular understanding of *CFTR* mutations by supplying structural pieces of information to design new approaches for the pharmacology dynamic even if the different drugs proposed were obtained by high-throughput screening (Callebaut et al., 2017). Till date, two CFTR-directed molecule classes have been developed: “potentiator” compounds increasing mutated CFTR activity at the cell surface and, “corrector” drugs improving altered protein processing and trafﬁcking to the cell surface (Wainwright et al., 2015; Rowe et al., 2017; Davies et al., 2018) (**Figure 6**). The first generation of the compounds has either been limited to a few patients with specific mutations (Ivacaftor) or was addressed to a larger group (Orkambi) and demonstrated moderate effects in CF (Mayer, 2016). For this reason, the U.K. National Institute for Health and Care Excellence (NICE) issued a draft guidance against recommending Orkambi. Recently, the FDA has approved an auspicious combination of molecules (Elexacaftor–Tezacaftor–Ivacaftor called Trikafta) to restore the function of p.Phe508del CFTR protein in CF patients even if patients had a single p.Phe508del allele. The combination of drugs relative to the control resulted in a percentage of predicted FEV1 that was more than 14 points higher and a rate of pulmonary exacerbations that was 60% lower through 24 weeks of treatment (Keating et al., 2018; Middleton et al., 2019). Unfortunately, a few pieces of information are available for the inflammatory aspects of these treatments. Although, recent evidence showed that the inflammation and lung status hampers these medications and can hinder their effects. Only one article has demonstrated that CFTR modulators can reduce excessive pro-inflammatory response following LPS (lipopolysaccharide) stimulation of CF monocytes (Jarosz-Griffiths et al., 2020). Moreover, in this article, the authors have also demonstrated that IL-8, IL-1β, and TNF-α (Tumor necrosis factor-α) decreased significantly in the serum of CF patients treated with Ivacaftor and Tezacaftor treatment. It is not known whether the observed effects are due to the restoration of Cl^-^ efflux, GSH (glutathione), or CFTR protein interactions present at the membrane.

Other new classes of mutation are in development, such as CFTR amplifiers, CFTR stabilizers, and read-through agents (**Figure 6**). CFTR amplifiers upregulate the expression, and indirectly, the activity of mutant CFTR. PTI-428 and PTI-CH are the two amplifiers who seem promising in pre-clinical and clinical studies. PTI-428 can enhance lung function in CF patients receiving Orkambi with no significant adverse effects. CFTR stabilizer as Cavosonstat inhibits the enzyme that is involved in regulating how much CFTR protein is present at the cell surface (Donaldson et al., 2017). It could potentially increase the benefits of other medications that target the CFTR function. Read-through drugs can help the ribosome skip over the early stop sequence in order to read the mRNA remaining information and generate CFTR protein. These therapies may be of interest to class I mutations where there is no production of mRNA or CFTR protein. Ataluren was developed as a potential treatment for these mutations, but its development was terminated due to failed clinical trial outcomes (Shoseyov et al., 2016).

This approach needs to be completed in the future evaluation of CF trials to understand the effects better and investigate the mechanism complex. It can be assumed that earlier treatment using these drugs may avoid structural damages and give rise to more efficient and prolonged results. We can imagine that the improvement of various dysregulated parameters will have long-term effects on the inflammation present in CF patients, even if indirectly. A recent article has highlighted that by Tobramycin or the AMP, 6K-F17 could restore the effects of Orkambi on p.Phe508del-CFTR protein, suggesting a significant role of infection in the CF pathology (Laselva et al., 2020). Furthermore, using this approach, they have demonstrated that the active AMP can down-regulate the expression of pro-inflammatory cytokines like IL-8, IL-6, and TNF-α in p.Phe508del-CFTR human BECs (Laselva et al., 2020).

Some exciting improvements in chloride efflux have been demonstrated using *S*ildenafil, a phosphodiesterase type 5 (PDE5) inhibitor. This drug recues p.Phe508del-CFTR trafficking *in vitro* experiments and decreases sputum elastase activity and, consequently, the inflammatory process (Lubamba et al., 2011; Taylor-Cousar et al., 2015). In parallel to Vertex’s studies, many other companies are interested in similar approaches to develop CFTR modulators that either restore the CFTR protein to the membrane or activate it (**Figure 5**). This research work has been essential over the last ten years, and many other molecules are currently being evaluated and at a different stage.

More recently, another promising strategy has been proposed to modulate post-transcriptionally activity of CFTR regulated by acting through miRNA. Distinct groups have proved that wild-type and mutated p.Phe508del human CFTR is regulated by miR-101-3p, miR-145-5p, miR-223-3p, miR-494-3p,and miR-509-3p (Glasgow et al., 2018). The approaches to inhibit the effect of these miRNAs have demonstrated an increase in CFTR protein expression and activity in BECs (De Santi et al., 2020). This approach is exciting, but further researches are needed to understand the subtility of this regulation better.

#### ENaC Channel

Since CFTR negatively regulates the activity of the ENaC sodium channel, different strategies have been proposed to decrease its activity. The first proposed molecule was Amiloride, which acts as a potassium-sparing diuretic, showing some benefit in both animal studies and clinical trials. Unfortunately, its efficacy was limited due to its short half-life (Zhou et al., 2008). This approach was repeated with the use of a new ENaC blocker called AZD5634 from AstraZeneca and BI1265162 from Boehringer Ingelheim. A phase Ib study and a phase II study to test, respectively, the safety and effectiveness of AZD5634 and BI 1265162 are underway in CF adults. Nowadays, a more recent and exciting approach, using aerosol antisense oligonucleotide (ASO) targeting ENaC mRNA (Ionis ENAC 2.5Rx), has demonstrated some interesting and impressive results on mice by restoring inflammation and inhibiting ENaC activity (Crosby et al., 2017). A first clinical study with this therapy is currently ongoing.

In the same way, Arrowhead asks to open a phase I/II trial into inhaled small interference RNA (iRNA) therapy. The drug, called ARO-ENaC, is an investigational RNA therapy designed to lower the production of the epithelial sodium channel alpha subunit (αENaC) in the lungs of CF patients. ARO-ENaC is an iRNA molecule intending to block the production of ENaC channels. It works by targeting and destroying the αENaC mRNA molecules, which are genetic messengers that carry the necessary information for making αENaC proteins and consequently ENaC activity.

#### ANO1 Channel

Since functional CFTR rescue remains limited, with mutation-dependent effects, alternative strategies have been suggested to compensate for the CFTR deficiency and were proposed as a potential CF therapeutic target. Such a strategy was the stimulation of calcium-activated chloride channels (CaCCs) such as the Anoctamin 1 channel (ANO1) (**Figure 3**). In the nineties, Knowles *et al.* have discovered that adenosine ‘5’-triphosphate and uridine-5’-triphosphate stimulated Cl^-^ secretion in both standards and CF respiratory epithelial, offering a potential by-pass mechanism for defective CFTR (Knowles et al., 1991). These activators transduce a signal through P2Y2 receptors that lead to the release of intracellular calcium and activate the CaCCs. An analog called Denufosol was developed. Different studies have demonstrated that this drug can increase Cl^-^ secretion through a CaCC, inhibit sodium absorption *via* the epithelial sodium channel called ENaC, and stimulate epithelial ciliary beat frequency (Accurso et al., 2011). Based on these data, ‘Denufosol’ clinical trials begun in 2001 using a wet nebulization direct airway delivery approach. Unfortunately, the last phase III had failed to demonstrate any benefit, and the project was dropped, but the idea of developing this approach remained (Moss, 2013). At the time of this study, CaCCs were poorly known. Their identity remained elusive for over 20 years until 2008 (Nilius and Droogmans, 2003; Caputo et al., 2008; Schroeder et al., 2008; Yang et al., 2008). When ANO1, the principal CaCC present in the airways, was identified in 2008, it allowed for more targeted approaches. Attractively, ANO1 channel has, at the apical membrane of epithelial cells, the same expression pattern as CFTR channels, and this protein was shown to be essential in the activity of CFTR as a chloride channel (Benedetto et al., 2017; Benedetto et al., 2019). Besides, ANO1 is implicated in HCO_3_ different permeability, proliferation, wound healing, inﬂammation, and its expression decreased in CF patients (Veit et al., 2012; Jung et al., 2013; Ruffin et al., 2013). Moreover, a recent article highlighted that ANO1 inhibition decreased ASL height. The authors have also demonstrated that ANO1 is not required for MUC5AC expression, the main protein of the mucus (Simoes et al., 2019). For this reason, a novel ANO1 potentiator was developed (ETX001), and airway epithelial function and mucus transport were evaluated in the human cells and animal models. This approach confirmed previous results and demonstrated that this drug could increase epithelial fluid secretion and enhance mucus clearance (Danahay et al., 2020).

Recently, our group has proposed a particular strategy using an ASO specific to ANO1 to reestablish ANO1 expression in the context of CF. This strategy “hijacks” the miRNA regulatory system and allows highly targeted effects. We have demonstrated that ASO-ANO1 could be used to inhibit the fixation of miR-9 on ANO1 mRNA by a target site blocker, and consequently to activate the alternative chloride channel to compensate CFTR Cl^-^ deficiency regardless of the mutation (Sonneville et al., 2017). We have also shown that with this strategy, we can improve tissue repair on cell lines but also on CF primary patient cells. We have likewise demonstrated that with this approach, we can activate mucociliary clearance on primary cells but also CF mice. Although we have not studied the effects of ANO1 modulation of inflammation, preliminary studies have already shown that activating ANO1 limits the secretion of IL-8 (Veit et al., 2012).

### Novel Anti-Cytokines Approaches

A pathophysiology pulmonary characteristic of CF is a severe neutrophil accumulation, which is correlated with high levels of pro-inflammatory cytokines (IL-8, IL-6, TNF-α), and low levels of anti-inflammatory mediators like IL-10 (Jacquot et al., 2008b). For numerous years, different approaches, as curcumin or vitamin D, have been proposed to limit IL-8 secretion and neutrophils influx (Gaggar et al., 2011; Olszowiec-Chlebna et al., 2019). Some pre-clinical data have demonstrated that using antibodies, like antibodies directed against intercellular adhesion molecule (ICAM)-1 and IL-8, could be a promising target. The most advanced therapy using SB-656933, an oral CXCR2 antagonist, was already tested in CF patients and has demonstrated along with safety some exciting results in the modulation of airway inflammation (Moss et al., 2013). However, another study using SCH527123 (MK-7123, Navarixin), a CXCR1/2 antagonist, was also attempted in chronic obstructive pulmonary disease (COPD) but was abandoned because of a severe decline in neutrophil number (Rennard et al., 2015). By contrast, a phase II clinical trial has already been carried out in patients with ulcerative colitis and demonstrated inhibition of ozone-induced airway inflammation in humans (Lazaar et al., 2011). Numerous other modulators of cytokines in the context of CF have been proposed, but only *in vitro* experiments have been performed (Lampronti et al., 2017; De Fenza et al., 2019). Cytokine modulation shows that cytokines have a significant role in limiting infections, although these approaches are confusing. A recent publication has highlighted the role of an IL-1 signaling pathway in sterile neutrophilic inflammation and mucus hypersecretion and has suggested that treatment with IL-1 receptor antagonist as Anakinra could be promising to prevent lung inflammation (Balazs and Mall, 2019).

The possibility of increasing gene expression and protein activity by the use of ASO has become more and more promising in the last years. However, long-term eﬃcacy, safe delivery, and side eﬀects of long-term treatment must be evaluated in order to be applied in patients with CF (Bardin et al., 2018b; Vencken et al., 2019). Fabbri *et al.* have developed this original concept by modulating the IL-8 expression by increasing miR-93 in BECs during *P. aeruginosa* infection (Fabbri et al., 2014). More recent results have highlighted that other miRNA involved in CF pathology, like miR-199a-3p or miR-636, could be targeted to control the CF lung inflammatory process (Bardin et al., 2018a; Bardin et al., 2019). Other interesting approaches have been performed to modulate the cascade of inflammation targeting NFκB activity by using, for example, Angelicin derived from different angiosperms or Sulindac, an NSAID (Rocca et al., 2016). Unfortunately, these approaches are not specific, and the risk of side effects remains high.

### New Development in Antibiotic Approaches

#### “Synthetic” Antibiotics

In CF, antibiotics are utilized for various applications, such as initial infection prevention, eradication (for early infection), control (for chronic infection), and finally, pulmonary exacerbations treatment. The antibiotics are given in three different primary ways: oral, inhalation, or intravenous. The choice of antibiotics depends on the nature of the pathogen to be eliminated, the age of the patient, and the nature of other pathogens present such as *H. influenza*, *S. aureus*, or *P. aeruginosa* infections.

*P. aeruginosa* is an opportunistic Gram-negative pathogen and is one of the main reasons for morbidity and mortality in CF and immunosuppressed patients. In order to eradicate new *P. aeruginosa* infections, antibiotic regimens are now a care standard around the world. Different groups assessed the effectiveness of inhaled Tobramycin, Aztreonam, and Colistin as well as oral Ciproﬂoxacin in eradicating new *P. aeruginosa* infection (Waters, 2018; Pang et al., 2019), although *P. aeruginosa* eradication is now much more challenging as a result of its impressive capability to resist antibiotics. These organisms become embedded in an exopolysaccharide biofilm, which protects the organism from phagocytosis and reduces the efficacy of anti-microbial drugs (Doring, 2010). Once this change has occurred, the mucoid *P. aeruginosa* could acquire multi-drug resistance, and this bacterium is virtually impossible to eradicate (Southern et al., 2012). If the *P. aeruginosa* infection cannot be cleared, the affected person is faced with an increased treatment burden, accelerated decline in lung function, increased symptom severity, and increased mortality (Nixon et al., 2002).

Recently, there has been a growing number of “new” antibiotics, of different classes and formulations, for pulmonary infection treatments in CF patients (Waters and Smyth, 2015). In order to limit toxicity and reduce side effects while directly targeting the lungs, many studies took an interest in using aerosols as a method of administration. In this frame of mind, Levofloxacin was developed for CF patients to target *P. aeruginosa* infections (Chirgwin et al., 2019; Epps et al., 2019). This drug, derived from the fluoroquinolone family, inhibits topoisomerases, which is essential for the synthesis of bacterial DNA. In the same way, inhaled Zitreonam is now available to treat *P. aeruginosa* infections in CF patients. Although its aerosolized formulation was proven to be beneficial, the formulation for intravenous injections induces significant lung inflammation, which has limited its use. Another example of the existing improvement of drugs is Tobramycin, presented as a dry powder. Inhaled tobramycin provides, in less than 5 minutes, a rapid action directly at the site of the lung infection.

In order to increase the efficacy of *P. aeruginosa* eradication and have a less often resistance development in comparison to the existing “classical” antibiotics, recent *P. aeruginosa* suggested treatment is the use of a combination of antibiotics and the development of new ones. Also, they can be associated with an alternative strategy such as EDTA (Respirion) or inhaled glycopolymer (SNP113).

Thus, a new carbapenem antibiotic called Doripenem has been developed with wide spectrum activity against bacteria through bacterial cell wall synthesis inhibition. Different authors have shown *in vitro* that this molecule has more significant activity than other antibiotics of the same family on strains isolated from CF patients (Traczewski and Brown, 2006; Riera et al., 2011). A clinical phase III study showed that patients infected with *P. aeruginosa* and treated with Doripenem had higher recovery rates in comparison to Imipenem-treated patients but, no clinical trial with CF patients is in progress (Chastre et al., 2008). In the same way, Plazomicin (a semisynthetic aminoglycoside) and POL7001 (a protein epitope mimetic) came out as an interesting strategy against *P. aeruginosa* (Cigana et al., 2016). These drugs have demonstrated *in vitro* some exciting effects on the multidrug-resistant *P. aeruginosa* isolated from CF patients (Cigana et al., 2016).

#### “Natural” Approaches

For many years an original approach using bacteriophages has been advanced. Bacteriophages were discovered in 1915 and can kill bacteria by causing lysis (Summers, 2001). Bacteriophage therapy was applied extensively in the 1930s and 1940s before antibiotics, and it is still being used in Eastern Europe. Nevertheless, after antibiotics became broadly accessible, phage therapy was renounced in Western countries. Many phages can target *P. aeruginosa* and have demonstrated some exciting effects on mice by decreasing the bacteria burden in the lungs or preventing infection (Morello et al., 2011). Even if clinical studies have shown relative effectiveness, treatments using phages remain negligible so far. Various reasons have limited the treatments with bacteriophages. The idea of introducing a living organism into the body is difficult to accept and remains an important psychological barrier. Moreover, early tests showed that the preparation generated impurities and that these preparations were not very stable (Morello et al., 2011). Although the use of phages in combination with quorum sensing inhibitors seems interesting, this approach remains marginal (Pang et al., 2019), and only a phase Ib/II trial is planned to test the safety and tolerability of AP-PA02 in adults with CF. AP-PA02 is a type of phage intended to control *P. aeruginosa* infections in CF patients. In *in vitro* studies, AP-PA02 can kill more than 80% of *P. aeruginosa* strains from CF people, and some first results are encouraging (Law et al., 2019).

Another “natural strategy” is inhaled nitric oxide (NO) for which an initial phase II study is underway. NO is a gas derived from nitrogen with anti-microbial properties. Some *in vivo* studies have validated this approach to eradicate the infection and to decrease mucus viscoelastic (Rouillard et al., 2020).

In the late 1970s, various studies showed that iron played an essential role in bacterial growth and was involved in particular in DNA replication, energy production, and pathogen-host interaction (Payne and Finkelstein, 1978). Recent results demonstrated that the iron content of human sputum is considerably high in CF, which facilitates chronic infections in the lungs of CF patients (Reid et al., 2007). These observations resulted in the development of novel therapeutic strategies in order to limit the amount of iron present in the airways. Gallium is a compound that shares the same properties with iron. It has demonstrated *in vitro* and *in vivo* anti-Pseudomonas properties (Tovar-García et al., 2020). The FDA has already approved the intravenous administration of Gallium. Clinical studies, in phase II for intravenous and a phase I for an inhaled strategy, are ongoing to evaluate its efficiency in treating *P. aeruginosa* infections in CF patients (Tovar-García et al., 2020).

**Figure 7:**
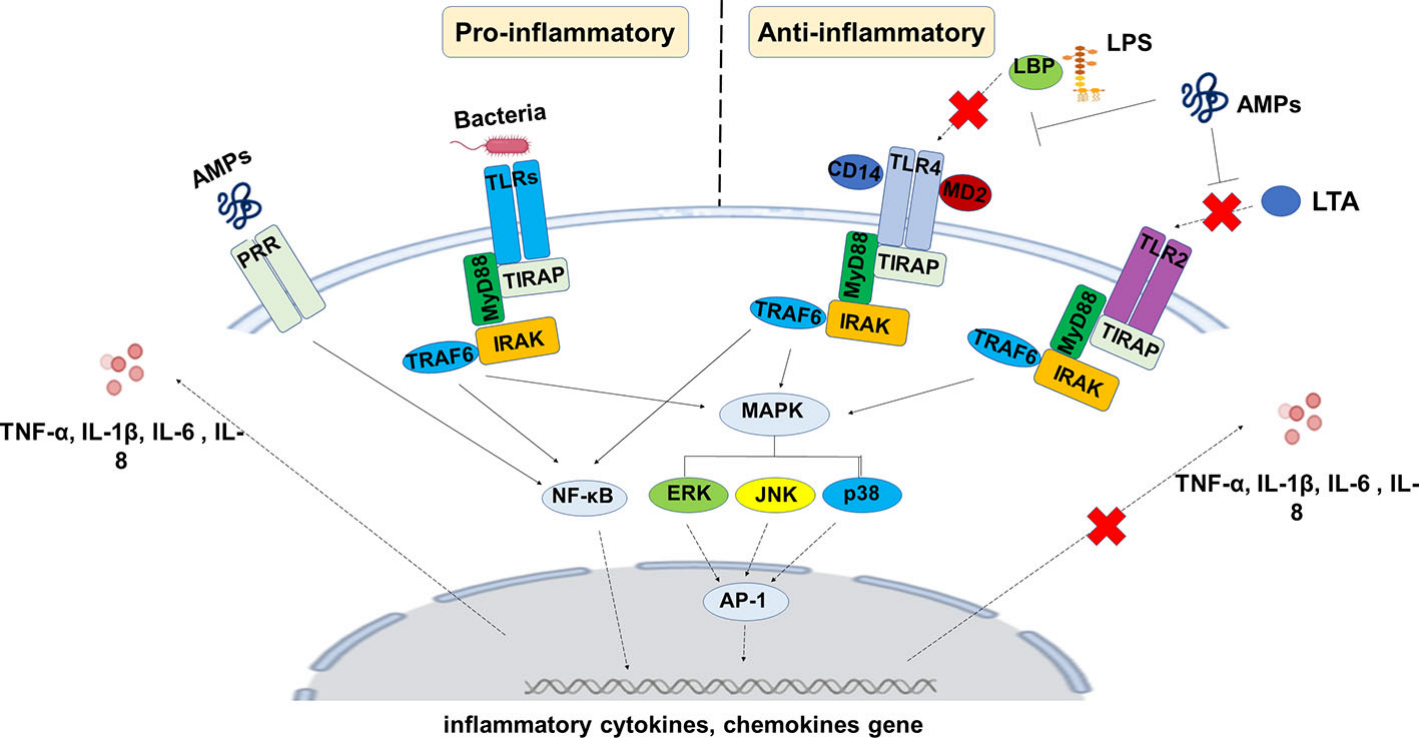
General mechanisms by which AMPs exert anti-inflammatory actions on host cells. AMPs can bind to bacterial virulence factors such as LPS or LTA and prevent their interactions with host cells. AMPs are also able to interfere with host cell signaling pathways involved in the inflammatory reaction. The overall consequence is that AMPs reduce the production of inflammatory mediators by these cells that may help in the resolution of inflammation.

During the last decades, AMPs naturally emerged as a potential therapy to cure infections with antibiotic resistance, in CF included. Treatments of bacterial infections by antibiotics result in a worldwide spread of dissemination of antibiotic resistance, both in the community and clinical settings. Besides, the development of new antibiotics is costly and time-consuming. It is hence of great importance to note that AMPs can treat methicillin-resistant *S. aureus* and multidrug-resistant *P. aeruginosa* that are resistant to conventional antibiotics (Geitani et al., 2019). Studies showed that treatments of antibiotic-resistant bacterial strains with AMPs were associated with almost no induced resistance to AMPs, which may encourage their use as potential replacement therapy for antibiotics. AMPs can exert anti-inflammatory actions either by suppressing the production of pro-inflammatory cytokines or by stimulating that of anti-inflammatory cytokines by host cells (**Figure 7**). Cathelicidin LL-37 (one of the most studied AMPs) enhances the production of the anti-inflammatory cytokine IL-1R by the human peripheral blood-derived mononuclear cells and macrophages (Choi et al., 2014), and similar results were observed with LL-37 and beta-defensin-3 (hBD-3) (Mookherjee et al., 2009; Smithrithee et al., 2015). Besides their direct actions on host cells involved in the initiation/modulation of inflammation, a number of AMPs, such as LL-37, Magainin-2, and bactericidal-permeability-increasing (BPI), can neutralize the activity of bacterial toxins such as LPS, thus participating in maintaining a balance between pro- and anti-inflammatory cytokines (Sun and Shang, 2015; Skovbakke and Franzyk, 2017).

Most of the reported studies in the field have focused on the roles of AMPs in the modulation of cytokine production. However, cytokines are only the tip of the iceberg in the inflammatory process, and other mediators of inflammation, such as eicosanoids, deserve to be investigated to identify their relative role in the modulation of inflammation by AMPs. Indeed, studies have reported that AMPs such as LL-37 modulates the production of eicosanoids, including leukotriene B4 (LTB4) and thromboxane A2 (TXA2) by macrophages (Agier et al., 2015). TXB2 and LTB4 are metabolites of arachidonic acid conversion by COX and lipoxygenase (LOX), respectively, and known to induce platelet aggregation and neutrophils recruitment at the site of infection (Yeung and Holinstat, 2011). It has been shown that LL-37 AMP blocks the expression of pro-inflammatory pathways involved, such as NF-κB in the presence of LPS (Agier et al., 2015). However, further studies are awaited to decipher the importance of the AMPs/eicosanoids network in the inflammatory reaction and potential implication in inflammatory diseases such as COPD, asthma, and CF. Similar anti-inflammatory effects were observed with WALK11.3 (an AMP with amphipathic helical conformation) in the mouse alveolar macrophage cell line RAW264.7 (Shim et al., 2015). They revealed the ability of this peptide to inhibit the expression of several inflammatory mediators, including NO, COX-derived metabolites, IL-1β, IL-6, interferon (IFN)-β, and TNF-α (**Figure 7**). The chicken cathelicidin-2 (CATH-2), the known ortholog of the human LL-37, has been shown to reduce inflammation in parallel to its anti-microbial activity against *P. aeruginosa*-resistant strains from CF patients (Banaschewski et al., 2017). The ability of CATH-2 to downregulate inflammation occurred through the anti-microbial-independent process, as this down-regulation was observed by silencing the inflammatory response that arises from killed bacteria. It is now clear that AMPs play a key role in host defense toward infectious by invading pathogens and represent a potential therapeutic tool to control infections by antibiotic-resistant bacterial strains. They also have the potential to protect the host from harmful inflammation that may result from these infections. Drug design and structure-relationship studies will greatly improve our knowledge of AMPs and the relative importance of their bactericidal *vs* anti-inflammatory functions, which will be of great help to optimize their potential therapeutic use in disease characterized by both chronic infection and inflammation such as CF.

All these data suggested that AMPs could be useful for clinical applications in the view of the protective function against pathogens. A series of clinical trials have started mostly in the pediatric population, and some compounds have been used as topical treatments but not known in the CF context. Different AMPs are under evaluation for the treatment of acute skin infection as Bralicidin, Omiganan, LTC109 (phase II clinical trial), or Pexiganam (phase III clinical trial). Other strategies and applications are currently under study. For example, in sepsis, Talactoferrin was tested by systemic injection in phase II clinical study (Guntupalli et al., 2013). Initial results showed a significant decrease in mortality after 28 days of treatment. However, phase II/III oral Talactoferrin was stopped for problems of safety and efficacy (Vincent et al., 2015). In the case of meningococcemia, rBPI21 pre-clinical trial has demonstrated some anti-bacterial and anti-LPS effects. Encouraging results led to the initiation of a phase III study in children with severe meningococcal sepsis (Giroir et al., 2001). The study outcome showed a reduction in complications with a shorter hospitalization also suggests the possibility to treat with rBPI21 other patients, including CF. The therapeutic applications of *P. aeruginosa* have been summarized in a recent publication (Magrone et al., 2018). An alternative therapeutic pathway for the use of AMPs has been envisaged by indirectly promoting their expression through the use of natural compounds. Several compounds have been identified as the use of Apigenin to enhance the expression and activity of β-3 defensin and cathelicidin in mice (Hou et al., 2013). Similar effects have been observed with vitamin D on *in vitro* studies to increase β-2 defensins and LL-37 on keratinocytes (Kim et al., 2009).

The use of natural or synthetic antibiotics can have a significant influence on the emergence of new pathogens. It is well established now that microbiota composition and dynamic impact the host immunity, health, and diseases (Belkaid and Hand, 2014). However, a new concept is now progressively emerging, suggesting that the innate immune response of the host can also modulate, at least in part *via* AMPs, the microbiota composition. For example, recent studies reported the involvement of sPLA2-IIA in the selection of species in pathologies characterized by polymicrobial infections such as CF. *P. aeruginosa* is known to progressively colonize CF airways to become the dominant pathogen at later stages of CF. This pathogen induces the production by CF airways of sPLA2-IIA, which in turn eradicate *S. aureus*, therefore helping in its gradual elimination from CF airways and its substitution by *P. aeruginosa* (Pernet et al., 2014). This effect is mostly due to the intrinsic resistance of *P. aeruginosa* and high susceptibility of *S. aureus* to sPLA2-IIA, respectively. Finally, it emerges that AMPs represent valid substitutes of antibiotics when a condition of antibiotic resistance is established.

### Alternative Strategies

#### Anti-Proteases

CF “anti-protease therapies” can be separated into two separate groups of drugs: some to increase anti-protease and some to inhibit protease expression. CFTR is an essential apical GSH transporter in the lung, and can indirectly participate in the inflammatory process by reducing oxidative stress. Evidence supporting the occurrence of oxidative stress in CF is established and extensively described (Galli et al., 2012; Causer et al., 2020). Some interesting works have demonstrated that oxidative stress could suppress CFTR expression (Cantin et al., 2006). Oxidative stress has a major role in the development of lung pathology in CF children and will, in addition to having a role in lung remodeling, have a role in the pulmonary microbiota (Shi et al., 2019). A recent metanalysis has positively correlated the expression of antioxidants with body mass index and lung function in CF (Causer et al., 2020). The malabsorption of nutrients with antioxidants properties in CF, participate in the imbalance in favor of oxidative stress and disrupt redox signaling, and, finally, molecular damages even if some data appears to be conflicting (Shamseer et al., 2010; Siwamogsatham et al., 2014). Therefore, multiple studies have been carried out to check the anti-protease supplementation in CF (Galli et al., 2012). Some studies have focused on especially serine proteases *via* two distinct administration routes: aerosolized and intravenously (McKelvey et al., 2020). In CF, exocrine pancreatic insufficiency and reduced bile acids induce critical antioxidants malabsorption, including carotenoids (β-carotene), tocopherols (vitamin E), coenzyme Q10, and selenium. Supplementation of antioxidant micronutrients (vitamin E, C, D, β-carotene, and selenium) may, therefore, potentially help maintain an oxidant-antioxidant balance, and this aspect has been extensively reviewed (Sagel et al., 2011; Ciofu et al., 2019). In the same approach, LAU-7b, an oral drug, is a derived form related to vitamin A. This compound can reduce the lung inflammatory response of CF people. In parallel, a phase II clinical study to test the effectiveness and safety of LAU-7b in CF patients is underway (Lands and Stanojevic, 2016). LAU-7b, also called, Fenretidine, work to increase docosahexaenoic acid (DHA) and consequently CER concentration. Some authors supported that the decrease of CER concentration contributes to the persistent bacterial infection and the constitutive MAP kinases and NFκB activation (Guilbault et al., 2008; Guilbault et al., 2009).

Human α-1 antitrypsin (A1AT) is still the most studied drug by far. Different clinical trials were already achieved. An inhaled α1-proteinase inhibitor is known to reduce NE burden in some patients with CF. A phase I in non-CF bronchiectasis and an IIa clinical study with puriﬁed A1AT products given through inhalation in CF subjects were just finalized and have demonstrated safety and efficacy (Gaggar et al., 2016; Watz et al., 2019). In the conclusion of the second study, the daily α-1 hydrophobic chromatography process delivered for three weeks was safe, well-tolerated, and effective in raising the α1-PI levels in the sputum of subjects with CF. However, the effects were transient and difficult to predict due to the proteases’ variability in CF patients’ lungs. The administration by airway routeway effectively increased the concentration of A1AT in sputum. The current study was not powered to assess changes in FEV1 or biomarkers in sputum, and further clinical are needed.

In parallel, A1AT gene therapy is emerging. Some recent data have demonstrated encouraging results in the inhibition of miRNA, which targets the A1AT gene called SERPINA1 (Hunt et al., 2020). This strategy aims to by-pass protein regulation systems of the most abundant inhibitor of NE in the airways. It is an alternative to the delivery of recombinant by using miRNA-targeted therapies. It was found that dual miRNA and adeno-associated viral (AAV)-based therapy engendered the long-term knockdown of circulating Z-A1AT and could be a new strategy in CF (Mueller et al., 2012). This approach was fully described in a review published (Hunt et al., 2020). The other approach is to directly activate SERPINA1 using gene therapy by using viral vectors like retrovirus or adenovirus, but numerous side effects have been observed (Gregory et al., 2011). Their use remains challenging, especially in the CF field.

Another strategy proposed is to use serine protease inhibitors such as secretory leukoprotease inhibitor (SLPI) which act locally to maintain a protease/anti-protease balance, thereby preventing protease-mediated tissue destruction. SLPI is a well-characterized member of the trapping gene family of proteins and is produced by respiratory tract epithelial cells and phagocytic neutrophils. Different approaches have been proposed to increase the anti-protease activity by nebulizing SLPI, but the efficacy is currently being evaluated alone or in association with other strategies (McElvaney et al., 1993; Quabius et al., 2017). Currently, novel protease inhibitor drugs, which have promising interest in the CF context, are in development (DX-890, AZD9668, POL6014, Grifols T6006-201) in order to improve their resistance against inactivation.

Promoting tissue repair represents another strategy by focusing on the proteins involved. Matrix metalloproteinases (MMP) are a group of distinct metalloendopeptidase enzymes that regulate various inﬂammatory and repair processes. They are either secreted or anchored to the cell surface, and therefore their activity is directed against membrane proteins or extracellular proteins, including inﬂammatory mediators. In CF patients, different articles have demonstrated that MMP is upregulated in the sputum of patients and is related to tissue damage (Delacourt et al., 1995; Gaggar et al., 2011). Various pro-inflammatory cytokines induce them at the transcription level. They might include the activation of a diverse group of intracellular signaling pathways (such as p38 MAPK or ERK 1/2 MAPK), causing the activation of nuclear signaling factors like AP1, NFκB, and STAT (signal transducer and activator of transcription). Activation of MMP can be induced by proteases or oxidants and are controlled by tissue inhibitor of metalloproteases (TIMP). There have been increasing interests in modulating MMP activity to enhance disease outcomes, and different clinical studies are in progress with promising effects in CF. A phase II study with Andecaliximab/GS-5745 in CF adults is in progress and was tolerated in patients with ulcerative colitis or Crohn’s disease, and could be an exciting approach to control pulmonary degradation.

The approaches using protease inhibitors are very varied, and many studies are still in progress. Although these therapies have been shown to improve patients’ health outcomes, they can only be considered in combination with other therapeutic targets.

#### Eicosanoids Pathway

Alterations in the metabolism of fatty acids present in membrane lipids may have an essential role in the inflammatory CF pulmonary disease. The arachidonic acid (AA): docosahexaenoic acid (DHA) ratio in blood serum, pulmonary airways, and rectal biopsies are increased in CF patients with either pancreatic sufficiency or pancreatic insufficiency, as compared with healthy control subjects (Freedman et al., 2004). AA is stored in cell membranes and is released from membrane lipids by various PLA2 proteins. Some interesting studies have highlighted the implication of sPLA2 in the pathogenicity of CF mice showing that reduced CFTR expression increased cytosolic PLA2α (cPLA2α) activity. A review has summarized the state of the art of fatty acid metabolism in CF (Strandvik, 2010). These effects improved mucus secretion and accumulation in airway epithelia independent of CFTR chloride transport function (Medjane et al., 2005; Dif et al., 2010). Therefore, cPLA2α has been proposed as an appropriate new target for therapeutic intervention in CF (Dif et al., 2010). Small lipid mediators were produced in the course of inflammation resolution and generated varied responses, which are cell types and tissue speciﬁc. A large number of these molecules modulate inﬂammation processes and provide essential functions in chemoattraction, aggregation, and degranulation of inflammatory cells. They are also implicated in tissue and vascular permeability, bronchoconstriction, and mucus production. Some of the lipid mediators include lipoxins (LX), resolvins, protectins, and maresins, which are generated by the activity of lipoxygenases lipoxin A4 (LXA4).

Interestingly, inhibitors of the 12R-lipoxygenase have demonstrated an essential role in mucin expression. The inhibitors decreased MUC5AC mucin expression by the inhibition of the ERK/SP1 dependent mechanism (Garcia-Verdugo et al., 2012). LXA4 has been described as a significant signal for the inflammation resolution and is generated at a low level in the CF patients’ lungs. LXA4 and RvD1 activate a GPCR termed ALX/FPR2.

This pro-resolving receptor is recognized by annexin A1, an endogenous anti-inflammatory peptide. A recent article provides evidence that the miR-181b, overexpressed in CF cells, may be considered as a new strategy to decrease the anti-inflammatory process in CF *via* the normalization of the expression receptor-dependent LXA4 (Pierdomenico et al., 2017). The LXA4 inhalation consequences have been examined in a pilot study of asthmatic and healthy adult subjects. The drug was well-tolerated, and no harmful effect was observed (Christie et al., 1992). Some impressive results were observed in the topical treatment of infantile eczema (Wu et al., 2013). Together with data showing beneficial actions of LXA4 in the CF context, these results highlight additional studies to check whether the upregulation of the lipidic mediators’ pathway can be considered as an appropriate tactic to fight inflammation in CF patients (Higgins et al., 2015).

Similarly, the LTB4 produced by resting BECs has been proposed as a target. Inﬂammatory stimuli increase the production of LTB4 and might also contribute to progressive pulmonary destruction in CF. Bronchial epithelial LTB4 acts as a potent chemoattractant for neutrophils *via* the cell surface integrins upregulation. When these cells are activated and present at the site of inflammation, they can also participate in the secretion of LTB4. LTB4 synthesis includes lipid peroxidation by 5-lipoxygenase, and produce numerous ROS, and consequently, pro-inflammatory activation. A clinical trial with Montelukast (BIIL 284), a leukotriene receptor agonist, counting a small number of patients, has provided contentious results in CF patients. This therapy has demonstrated a notable decrease in serum eosinophil cationic protein levels and eosinophils without any signiﬁcant improvement in FEV1, and FEF25–75%. Also, this strategy has shown a signiﬁcant decrease in cough, serum, and sputum levels of eosinophil cationic protein and IL-8 chemokine. Moreover, an increase in serum and sputum levels of IL-10 has been observed. The trial was stopped early due to a significant increase in the risk of severe pulmonary events in patients receiving the active drug (Schmitt-Grohe and Zielen, 2005). A more recent drug, Acebilustat (CTX-4430), has been evaluated in CF patients. This drug has shown anti-inflammatory activity *via* the LTA4 hydrolase inhibition and LTB4 modulation. In two-phase I clinical trials, Acebilustat decreased the production of LTB4 and pro-inﬂammatory cytokines in healthy volunteers and CF patients, and in phase II, optimal dose and duration were identified for future studies (Elborn et al., 2017; Elborn et al., 2018).

#### Cannabinoid-Derived Drug

Ajulemic acid (JBT-101, Lenabasum) is a cannabinoid-derived molecule that preferably binds to the active CB2 receptor and is non-psychoactive. In some pre-clinical trials done on human lung cells obtained from CF patients, it was shown that Lenabasum stopped the production of both TNF-α and IL-6, two crucial pro-inflammatory cytokines that trigger inflammation. In phase I and II clinical trials, this drug demonstrated favorable safety and tolerability. Recently, a group has also shown significant efficacy in mice models of inflammation and fibrosis (Burstein, 2018). Therefore, phase II was initiated. It will be used to test safety, tolerability, pharmacokinetics, and efficacy of JBT-101 in 70 subjects ≥ 18 and < 65 years of age with documented CF. Treatment of CF patients with Lenabasum twice daily has been able to decrease the number of acute lung exacerbations as well as a reduction of inflammatory cells and mediators present in the sputum. A new clinical trial is undergoing and seeks to enroll more than 400 CF patients over numerous clinical sites.

### Mucus Therapies

In the lungs, the abnormal production of mucus has been assumed to participate actively in the early CF pathogenesis (Ehre et al., 2014). For many years, researchers and clinicians have been trying to understand the origin of mucus abnormalities and found mucoactive drugs molecules to control CF bronchial obstruction. Mucoactive drugs are regularly used as a therapeutic option and are defined by their activity as mucolytics, expectorants, and cough facilitating drug. The expectorants, such as hypertonic solution (HSS), increase the ASL layer and decrease mucus adhesiveness. Mucolytics, such as both N-acetylcysteine (NAC) and recombinant human DNase (rhDNase), reduce sputum viscosity. Medications such as inhaled mannitol, rhDNase (Dornase), and hypertonic HSS have proven efﬁcacy in CF and indirectly reduced inflammation in airways of CF patients (Tarrant et al., 2017). The low volume hypothesis would estimate that approaches increasing the ASL height will increase mucociliary clearance, and consequently reduce lung infection. In order to increase the ASL height and fluidity, an HSS (3 to 7% NaCl) has been proposed to treat CFTR deficiency for better mucociliary clearance. Recently, Wark & McDonald have performed a meta-analysis of 17 different clinical trials of HSS and concluded that, after four weeks, a small enhancement in the lung function was observed but was not sustained at 48 weeks. HSS might also have a little impact on improving life quality in adults (Wark and McDonald, 2018). New clinical trials are in progress in order to establish who may benefit most and whether this benefit is sustained in the longer term (https://www.cff.org/Trials/Finder).

In the same manner, a meta-analysis was performed with mannitol, which is a naturally occurring sugar alcohol. When inhaled mannitol creates a change in the osmotic gradient. It leads to water movement into the CF airway hydrating the ASL, and enhancing mucociliary clearance. In the different studies, there was no evidence showing that the mannitol treatment for over six months is related to an enhancement of lung function in CF patients compared to control (Nevitt et al., 2018). Recently, different groups have observed expression, biochemical and biophysical alterations of the mucous present in the airways of CF patients (Rhim et al., 2001). More, they observed that abnormal glycosylation of the airway mucins is associated with bacterial infection and inflammation. The effects of altered host mucin glycosylation affect *P. aeruginosa* adhesion and so pathogenicity. A review from Ventalakrishan et al. has extensively described this feature (Venkatakrishnan et al., 2013). Different therapeutic approaches have been proposed to correct this observation by using, for example, mannose-biding lectin, which recognizes bacterial glycoconjugates and participates in an effective defense against pathogens (Moller-Kristensen et al., 2006).

Another strategy used in CF is to disrupt the high DNA content present in the airway mucus of CF patients. DNA is a polyanion compound responsible for the viscosity and adhesiveness of the pulmonary secretions. DNA release and accumulation in ASL occur as a result of tissue destruction caused by inflammatory cells on bacteria and epithelial cells. The strategy is to use a recombinant human deoxyribonuclease I (rhDNase), an enzyme that selectively cleaves DNA, hence decreasing mucus viscosity (Puchelle et al., 1995). Nebulized rhDNase hydrolyzes extracellular DNA within the mucus and transforms it from an adhesive gel into a liquid form of fluid through dilution within minutes. In contrast to mannitol or HSS, rhDNase has shown some significant effects on the improvement of lung function of CF patients and is considered as an effective treatment for the liquefaction of viscous mucus in CF. However, individual responses are unpredictable (Yang and Montgomery, 2018).

The only approved reducing agent for human use is N-acetylcysteine (NAC), a well-known antioxidant GSH drug. This drug ameliorates the redox imbalance in neutrophils present in the blood and inhibits their recruitment in the airways of CF patients (Tirouvanziam et al., 2006). NAC is also used in CF as an aerosolized mucus solution to break down disulfide bonds between mucin proteins in order to fluidify mucus (Duijvestijn and Brand, 1999). Some evidence demonstrated that NAC has excellent anti-bacterial properties, the capacity to intervene with biofilm formation and, to disturb the adherence of respiratory pathogens to respiratory epithelial cells (Blasi et al., 2016). In CF patients, NAC has been proven to be safe at large doses with negligible interaction with other drugs. NAC was investigated in CF despite its partial effectiveness as an inhaled mucolytic agent because the extremely oxidizing CF airway environment consumes aerosolized antioxidants quickly (Tirouvanziam et al., 2006; Cantin et al., 2007). Finally, inhaled NAC is being used as a mucolytic drug in CF for several decades, although the positive results remain limited. Newer agents targeting other components of CF mucus are currently in development or clinical trials (NAC 40630) and exhibit an exciting effect on mucus (Blasi et al., 2016).

Another original approach is undergoing with OligoG CF-5/20. OligoG is an alginate oligosaccharide derived from natural seaweed. It is administrated using a dry powder inhaler and also developed as a liquid to use with a nebulizer. Studies have shown that this dry power drug is capable of reducing the mucus thickness in the lungs. In addition, this drug enhances the efficiency of antibiotics and may facilitate mucus clearance in CF patients. The drug could detach CF mucus by calcium chelation (Ermund et al., 2017). Initiated in 2018, phase II includes more than 120 patients from European and Australian sites. It aims to determine the optimal dose of OligoG and to describe long-term safety and efficacy, with FEV1 as a primary endpoint.

Recently, numerous articles have been published to describe new regulation mechanisms of the different proteins present in the mucus and especially on mucins expressed in the airways. The epigenetic regulation role of MUC5AC and MUC5B, the main mucins expressed in the airways, has been thoroughly researched in COPD and have highlighted the implication of methylation and miRNA. Different specific therapies are in progress to modulate the miRNA, and new treatment ways are in progress in CF (Bardin et al., 2018b).

## Conclusion

Although current anti-inflammatory drugs (corticosteroids and Ibuprofen) in CF patients have shown little effectiveness, the creation and improvement of new anti-inflammatory drugs for CF lungs has been overlooked for a long time. In the last decade, most of the research fields in CF therapy, have focused mainly on the discovery of new CFTR activators. Despite this, basic researches that are now in the evaluation phase have shown that new approaches could be very promising in resolving efficiently the CF lungs’ ongoing inflammatory vicious cycle. However, treatment complexity is challenging. Currently available treatments offered to CF patients certainly help reduce inflammation, but in indirect and non-specific pathways, by targeting the viscosity of the mucus, reducing infection, or activating Cl^-^ efflux. As the traditional approaches have shown their limitations, it seems essential to us that original work should continue in order to identify innovative approaches that would be more specific. The identification of critical druggable molecular targets to decrease inflammation is still an unsatisfied demand that needs numerous additional researches.

## Author Contributions

All authors: CM, PB, ZX, HC, LT, and OT have written and reviewed the manuscript.

## Funding

This review was funded in part by grants from Inserm, Sorbonne Université, Faculté des Sciences, Institut Pasteur, and the non-profit organization Vaincre la Mucoviscidose. CM received a Ph.D. grant from Vaincre la Mucoviscidose. ZX was founded by Yangzhou University.

## Conflict of Interest

The authors declare that the research was conducted in the absence of any commercial or financial relationships that could be construed as a potential conflict of interest.
